# Signal detection and safety analysis of three tyrosine kinase inhibitors for HER-2 positive breast cancer: a retrospective study based on the FAERS database

**DOI:** 10.3389/fphar.2025.1538881

**Published:** 2025-03-10

**Authors:** Xiting Tang, Chengliang Wang, Yanwei Li, Jing Tang, Guoping Zhang, Li Chen

**Affiliations:** ^1^ Department of Pharmacy, People’s Hospital of Ganzi Tibetan Autonomous Prefecture, Kangding, China; ^2^ Department of Pharmacy, West China Second University Hospital, Sichuan University, Chengdu, Sichuan, China; ^3^ Chinese Evidence-Based Medicine Center, West China Hospital, Sichuan University, Chengdu, Sichuan, China; ^4^ Department of Pharmacology, Faculty of Medicine and Nursing, University of the Basque Country, Leioa, Spain

**Keywords:** FAERS database, tyrosine kinase inhibitors (TKIs), HER-2 positive breast cancer, adverse event signals, safety analysis, disproportionality analysis (ROR, PRR)

## Abstract

**Objective:**

To identify adverse event (ADE) signals of three tyrosine kinase inhibitors (TKIs) (Tucatinib, Lapatinib, and Neratinib) used for HER-2 positive breast cancer by utilizing the FAERS database, and to analyze their safety profiles to provide references for clinical risk management.

**Methods:**

Data from the FAERS database spanning Q1 2015 to Q3 2024 were retrieved, including reports where Tucatinib, Lapatinib, or Neratinib was identified as the primary suspect drug. Disproportionality analysis (ROR, PRR) and the Comprehensive Standard method were employed to detect potential ADE signals. The distribution of ADEs across different System Organ Classifications (SOCs) was also analyzed.

**Results:**

A total of 7,848 ADE reports were analyzed, identifying 557 significant signals. The primary ADEs were concentrated in gastrointestinal disorders, general conditions, administration site reactions, and skin and subcutaneous tissue disorders. Neratinib exhibited higher gastrointestinal toxicity, Lapatinib was associated with notable skin toxicities, and Tucatinib showed specific adverse reactions linked to combination therapies.

**Conclusion:**

The three TKIs demonstrated distinct ADE signal profiles, with gastrointestinal, systemic, and skin toxicities being the major areas of concern. Future research should validate these findings and develop effective management strategies to enhance treatment safety and improve the quality of life for HER-2 positive breast cancer patients.

## 1 Introduction

HER-2 positive breast cancer is a subtype defined by the overexpression of the HER-2 gene, accounting for about 15%–20% of all breast cancer cases (Slamon et al., 1987). This subtype is associated with rapid tumor growth and a high metastatic potential, which has made it a primary focus in clinical research. In the past decade, targeted therapies, including tyrosine kinase inhibitors (TKIs), have significantly improved the treatment of HER-2 positive breast cancer. These drugs inhibit the kinase activity of the HER-2 receptor, thereby preventing tumor cell proliferation and metastasis, which leads to improved survival rates and treatment outcomes (Swain et al., 2015).

Despite these advancements, TKI therapies are associated with significant side effects that can reduce patients’ quality of life and increase the likelihood of treatment interruptions, potentially resulting in higher mortality rates (Saura et al., 2020; Murthy et al., 2020). Each drug is associated with distinct side effects: Neratinib is commonly linked to gastrointestinal issues, such as diarrhea and nausea; Lapatinib is associated with skin toxicities, including rashes and dryness; and Tucatinib combination therapies may lead to more widespread systemic effects, such as cardiac damage and liver dysfunction (Cameron et al., 2010; Xu et al., 2021).

While these findings are valuable, they are primarily derived from clinical trial data, which often have limitations, such as small sample sizes and strict inclusion criteria. These limitations hinder the generalization of the results to real-world clinical settings. Therefore, research using real-world data is crucial for gaining a more comprehensive understanding of the safety profiles of these drugs and for more accurately assessing their potential risks in diverse populations (Yazdani et al., 2020).

The FDA Adverse Event Reporting System (FAERS) is a global database that collects adverse drug event reports from across the globe. FAERS is a crucial tool for identifying potential safety risks of marketed drugs (Sakaeda et al., 2011). FAERS is particularly valuable because it includes data from diverse populations and clinical contexts, allowing for the identification of adverse reactions in real-world use. However, retrospective analyses of adverse event signals for HER-2 positive breast cancer TKIs remain scarce, particularly regarding their impact on different physiological systems and specific adverse event profiles (Harbeck et al., 2020).

This study aims to utilize the FAERS database to analyze adverse event signals for Tucatinib, Lapatinib, and Neratinib, focusing on gastrointestinal disorders, systemic effects, and skin toxicities (Sharma et al., 2022). By conducting a comprehensive analysis of the adverse events associated with these drugs, this study seeks to identify the primary safety profiles of each drug. This will provide valuable insights for clinical drug risk management and contribute to the development of personalized treatment strategies for HER-2 positive breast cancer (Modi et al., 2020).

## 2 Materials and methods

### 2.1 Data source

This study analyzed data from the FAERS database, which is updated quarterly and serves as a comprehensive repository of detailed post-marketing adverse event reports. However, as outlined by the FDA in their FAERS public dashboard description (FDA, 2024), the database has limitations related to data validity, including underreporting, voluntary reporting bias, and the potential for incomplete or inaccurate reports. These limitations should be considered when interpreting the findings of this study. The database provides detailed information on report counts, patient demographics (e.g., age and gender), and the severity of adverse drug events (ADEs). The FAERS database consists of seven key tables: patient demographics and administrative details (DEMO), drug information (DRUG), adverse reaction records (REAC), patient outcomes (OUTC), report sources (RPSR), therapy timelines (THER), indications for use or diagnoses (INDI), and deleted case records (DELETED).

### 2.2 Data processing

Data were retrieved from the FAERS database by querying the generic names “Tucatinib,” “Lapatinib,” and “Neratinib,” covering 39 quarters from Q1 2015 to Q3 2024. However, the FAERS database has certain limitations, including the possibility of underreporting and biases due to voluntary reporting, which may impact the generalizability and completeness of the data. Only reports where the target drug was identified as the primary suspect were included. Potential duplicates were removed using a deduplication process, necessitated by the quarterly updates of the database. In accordance with FDA guidelines (Hu et al., 2020), duplicates with identical CASEID values were resolved by retaining the most recent FDA_DT. If both CASEID and FDA_DT were identical, the record with the higher PRIMARYID was prioritized. Reports listed in the DELETED table were excluded from analysis. Data were imported and analyzed using MySQL 8.0.

### 2.3 Data standardization

The FAERS database uses the Medical Dictionary for Regulatory Activities (MedDRA) coding system to classify and standardize adverse event data. In this study, MedDRA version 27.0 preferred terms (PT) and system organ classifications (SOC) were utilized to standardize the descriptions of adverse drug events (ADEs) (Sakaeda et al., 2013; Omar et al., 2021).

### 2.4 Data analysis

The number of ADE reports identifying the target drug as the primary suspect was compiled. Potential ADE signals were identified using disproportionality analysis (Table 1) (Sakaeda et al., 2013; Luo et al., 2021). The Reporting Odds Ratio (ROR) and Comprehensive Standard (MHRA) methods were applied to calculate ROR, proportional reporting ratio (PRR), and chi-square (X^2^) values. To minimize false-positive signals, only values exceeding predefined thresholds were recognized as valid signals for PTs (Table 2) (Chen et al., 2022; Sakaeda et al., 2011; Jin et al., 2021). Higher values represent stronger signals, reflecting an increased likelihood of an association between the target drug and the ADE, although causality cannot be confirmed (Zhou et al., 2022). All statistical analyses and visualizations were conducted using Microsoft Excel and GraphPad Prism 8.

**TABLE 1:** Fourfold table of disproportional method.

Drug category	Number of target ADE reports	Number of other ADE reports	Total
Target Drug	a	b	a+b
Other Drugs	c	d	c + d
Total	a+c	b + d	N = a+b + c + d

**TABLE 2:** Formulas and thresholds of ROR and PRR methods.

Method	Formula	Threshold
ROR Method	$ROR=\frac{a/c}{b/d}$ $95\%CI=e^{\text{In}\left(ROR\right)\pm 1.96\sqrt{\left(\frac{1}{a}+\frac{1}{b}+\frac{1}{c}+\frac{1}{d}\right)}}$	a≥3,Lower bound of 95% CI forROR > 1Considered a valid signal
MHRA Method	$PRR=\frac{a/\left(a+b\right)}{c/\left(c+d\right)}$ $X^{2}=\frac{{\left(ad-bc\right)}^{2}\left(a+b+c+d\right)}{\left(a+b\right)\left(c+d\right)\left(a+c\right)\left(b+d\right)}$	a≥3,PRR≥2,X2≥4Indicative of a valid signal

## 3 Results

### 3.1 Basic information on ADE reports

This study analyzed 7,848 adverse event reports for Tucatinib, Lapatinib, and Neratinib obtained from the FAERS database, spanning Q1 2015 to Q3 2024. The findings indicated that the majority of adverse event reports involved female patients, while Neratinib had a notably higher proportion of cases with unspecified gender. Reports involving male patients were also observed. Most cases with available age information involved patients aged 18–60 years, although some reports included patients under 18. A substantial proportion of cases lacked age data. Most reports were submitted by healthcare professionals; however, Tucatinib and Lapatinib had a relatively higher proportion of non-professional submissions. Geographically, the United States accounted for the largest number of reports, followed by France, India, and China (see Table 3).

**TABLE 3:** Summary of Basic Information on TKI-Related ADE Reports

Information	Category	Reported Cases [n (%)]		
		Tucatinib	Lapatinib	Neratinib
Cases		2713	3503	1632
Gender	Male	94 (3.46%)	160 (4.57%)	2 (0.12%)
	Female	2441 (89.97%)	2927 (83.56%)	54 (3.31%)
	Unknown	178 (6.56%)	416 (11.88%)	1576 (96.57%)
Age Group	≤18	1 (0.04%)	3 (0.09%)	0 (0.00%)
	18∼60	562 (20.72%)	1060 (30.26%)	34 (2.08%)
	≥60	523 (19.28%)	795 (22.69%)	14 (0.86%)
	Unknown	1627 (59.97%)	1645 (46.96%)	1584 (97.06%)
Reporter	Healthcare Providers	1782 (65.68%)	1775 (50.67%)	1412 (86.52%)
	Non-Healthcare	929 (34.24%)	1626(46.42%)	214(13.11%)
	Unknown	2 (0.07%)	102 (2.91%)	6 (0.37%)
Top5 Reported Countries		US [2234 (82.34%)]	US [2229 (63.63%)]	US [1377 (84.38%)]
		FR [120 (4.42%)]	IN [106 (3.03%)]	CA [57 (3.49%)]
		GR [53 (1.95%)]	CN [93 (2.65%)]	AR [52 (3.19%)]
		DE [44 (1.62%)]	JP [91 (2.60%)]	GB [34 (2.08%)]
		ES [34 (1.25%)]	IT [73 (2.08%)]	DE [33 (2.02%)]

### 3.2 ADE signal detection results

This study identified 557 significant signals from the adverse event reports of 7,848 patients, with the top 20 preferred terms (PTs) ranked by descending Reporting Odds Ratio (ROR) (see Table 4). The identified adverse events varied substantially across the drugs. For Tucatinib, notable high-signal events included “Gamma ray therapy” (ROR: 1775.84) and “Congenital malformation of the lung and airway” (ROR: 496.87). Lapatinib showed significant associations with “HER2-positive breast cancer” (ROR: 89.93) and “Nail bed bleeding” (ROR: 72.67). For Neratinib, high signal intensities were observed for “Drug titration” (ROR: 4451.91) and “Breast reconstruction” (ROR: 209.00).

**TABLE 4:** Top 20 PTs of TKI-Related ADEs by Report Count and ROR.

Tucatinib				Lapatinib				Neratinib			
PT	Case (n)	ROR (95% Cl lower limit)	PRR (X^2^)	PT	Case (n)	ROR (95% Cl lower limit)	PRR (X^2^)	PT	Case (n)	ROR (95% Cl lower limit)	PRR (X^2^)
Gamma radiation therapy	5	1775.84 (639.19)	1772.57 (6523.15)	HER2 positive breast cancer	5	89.93 (37.03)	89.81 (429.06)	Drug titration	40	4451.91 (3026.26)	4342.82 (113760.31)
Congenital pulmonary airway malformation	3	496.87 (151.55)	496.32 (1348.15)	Nail bed bleeding	7	58.49 (31.30)	58.33 (555.03)	Breast reconstruction	5	209.00 (85.93)	208.37 (1006.46)
Congenital pulmonary airway malformation	3	496.87 (151.55)	496.32 (1348.15)	Onychalgia	10	38.01 (30.30)	37.20 (2688.06)	Breast cellulitis	3	154.03 (49.11)	153.75 (446.95)
Nail discomfort	3	186.32 (58.81)	186.12 (532.41)	Nail bed disorder	4	29.87 (22.09)	29.52 (1176.24)	Metastases to abdominal cavity	3	85.51 (27.39)	85.36 (247.56)
Brain tumour operation	4	164.31 (60.64)	164.07 (627.57)	Metastases to central nervous system	77	28.09 (10.50)	28.06 (103.61)	Breast cancer metastatic	35	52.61 (37.60)	51.51 (1723.38)
Hypertelorism	3	158.57 (50.20)	158.40 (454.71)	Colorectal cancer metastatic	9	25.84 (16.44)	25.71 (448.24)	Diarrhoea	1016	49.30 (44.61)	19.23 (18107.56)
Eating disorder symptom	9	152.96 (78.71)	152.45 (1313.81)	Breast cancer metastatic	43	24.50 (10.97)	24.46 (134.14)	Early satiety	4	41.88 (15.66)	41.78 (158.42)
Fingerprint loss	3	131.91 (41.89)	131.77 (379.26)	Metastases to skin	5	22.63 (16.13)	22.42 (692.05)	Emergency care	11	38.60 (21.30)	38.35 (398.31)
Tumour marker abnormal	7	116.88 (55.19)	116.58 (783.76)	Pleural thickening	4	19.91 (13.62)	19.76 (478.61)	Mastectomy	4	34.21 (12.80)	34.13 (128.12)
Radiotherapy	24	103.68 (69.09)	102.78 (2369.97)	Malignant ascites	3	18.64 (5.99)	18.63 (49.81)	Metastases to central nervous system	25	26.14 (17.60)	25.76 (593.47)
Craniotomy	5	97.50 (40.20)	97.32 (467.48)	Paronychia	19	25.00 (8.04)	24.95 (68.78)	Bladder spasm	3	25.00 (8.04)	24.95 (68.78)
Fluid replacement	5	97.50 (40.20)	97.32 (467.48)	Palmar-plantar erythrodysaesthesia syndrome	97	24.50 (10.98)	24.41 (134.34)	Drug titration error	6	24.50 (10.98)	24.41 (134.34)
Tumour marker decreased	3	96.17 (30.66)	96.06 (276.86)	Nail infection	6	23.31 (7.50)	23.27 (63.75)	Onycholysis	3	23.31 (7.50)	23.27 (63.75)
Ear malformation	3	73.43 (23.47)	73.35 (210.97)	Brain cancer metastatic	3	22.10 (10.51)	22.01 (140.06)	Breast cancer stage IV	7	22.10 (10.51)	22.01 (140.06)
Brain operation	21	69.93 (45.38)	69.39 (1396.18)	Brain neoplasm	34	21.42 (12.40)	21.26 (250.41)	Onychoclasis	13	21.42 (12.40)	21.26 (250.41)
Intracranial tumour haemorrhage	3	64.53 (20.65)	64.46 (185.02)	Breast cancer recurrent	10	19.20 (9.13)	19.12 (119.95)	Gastrointestinal sounds abnormal	7	19.20 (9.13)	19.12 (119.95)
Nose deformity	3	61.85 (19.80)	61.78 (177.19)	Nail disorder	27	19.13 (6.16)	19.10 (51.34)	Faeces pale	3	19.13 (6.16)	19.10 (51.34)
Blood bilirubin abnormal	9	59.36 (30.73)	59.16 (508.59)	Noninfective encephalitis	3	17.57 (8.76)	17.49 (124.12)	Dermatitis acneiform	8	17.57 (8.76)	17.49 (124.12)
Tumour excision	6	59.33 (26.51)	59.20 (339.29)	Breast neoplasm	3	17.50 (12.46)	17.16 (516.9)	Prescribed underdose	34	17.500 (12.46)	17.16 (516.90)
Product temperature excursion issue	34	57.01 (40.57)	56.31 (1826.69)	Oncologic complication	3	15.79 (11.18)	15.49 (446.95)	Blood potassium decreased	33	15.79 (11.18)	15.49 (446.95)

### 3.3 System organ class involvement in ADE signals

**FIGURE 1:**
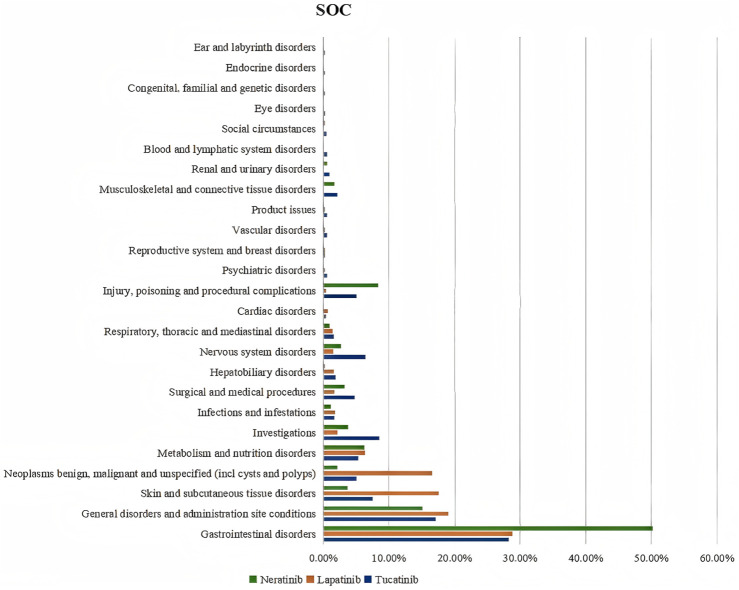
SOC occurrence of TKIs ADEs.

The three TKIs exhibited distinct distribution patterns across System Organ Classification (SOC) categories of adverse events. Tucatinib was associated with 23 SOCs, Lapatinib with 18 SOCs, and Neratinib with 20 SOCs. Adverse events were primarily concentrated in gastrointestinal disorders and general disorders, including administration site conditions. Gastrointestinal disorders accounted for 28.27% and 28.79% of adverse events for Tucatinib and Lapatinib, respectively, whereas Neratinib demonstrated a significantly higher proportion at 50.21%. Additionally, Lapatinib exhibited the highest percentage of adverse events related to skin and subcutaneous tissue disorders (17.58%), while Neratinib showed the highest proportion in injury, poisoning, and procedural complications (8.33%). Overall, adverse events predominantly concentrated in gastrointestinal disorders, general disorders (including administration site conditions), and skin and subcutaneous tissue disorders (see Figure 1).

#### 3.3.1 PT distribution in gastrointestinal disorders

**FIGURE 2:**
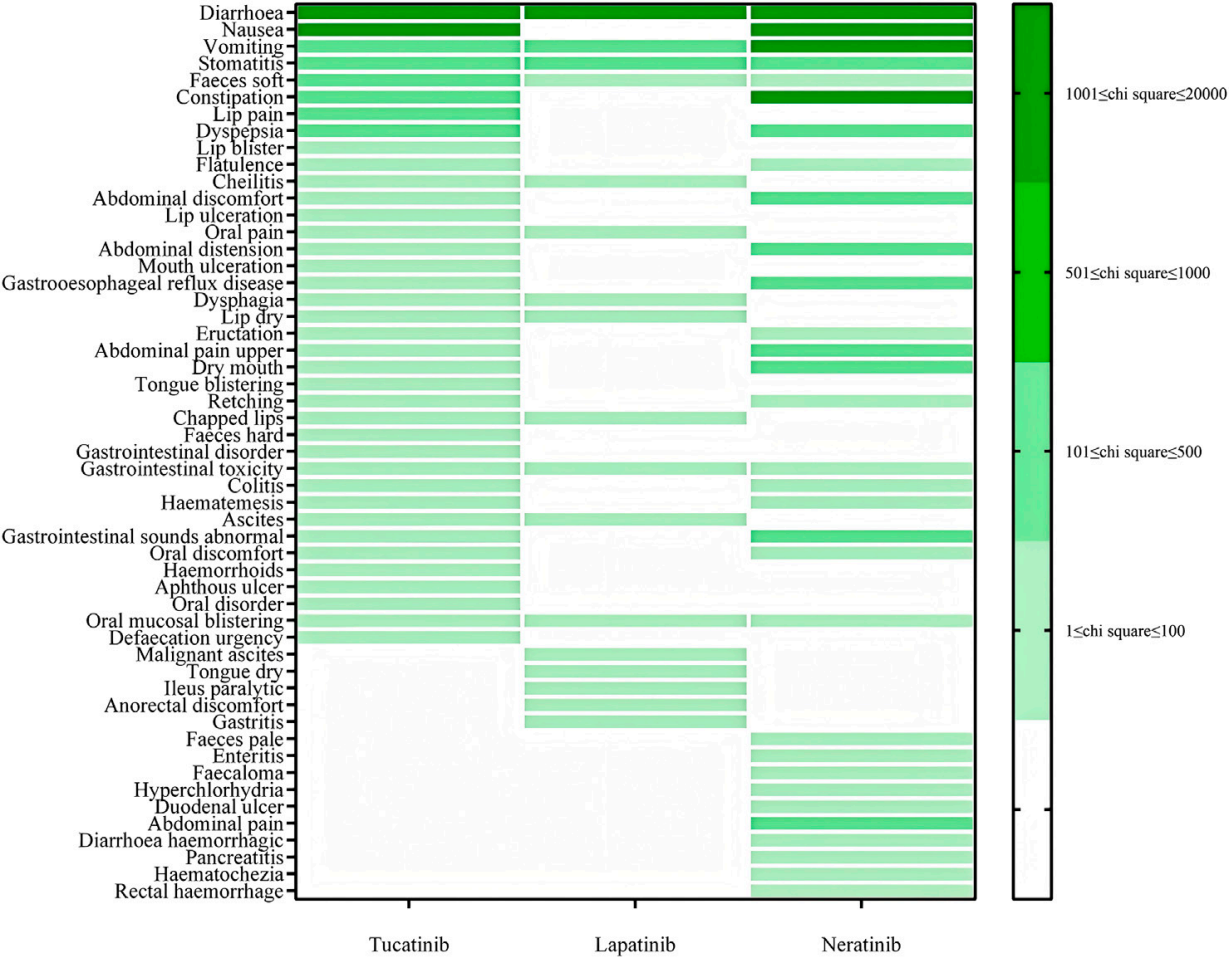
PT distribution for gastrointestinal disorders.

For gastrointestinal disorders, Tucatinib was most strongly associated with diarrhea (ROR: 6207.48), followed by nausea (ROR: 1249.54) and vomiting (ROR: 496.59). Lapatinib showed significant associations with diarrhea (ROR: 4449.35), stomatitis (ROR: 179.04), and vomiting (ROR: 155.14). Neratinib exhibited a high prevalence of gastrointestinal events, particularly diarrhea (ROR: 18107.56), nausea (ROR: 2593.08), constipation (ROR: 2585.67), and vomiting (ROR: 1169.06) (see Figure 2).

#### 3.3.2 PT distribution in general disorders and administration site conditions

**FIGURE 3:**
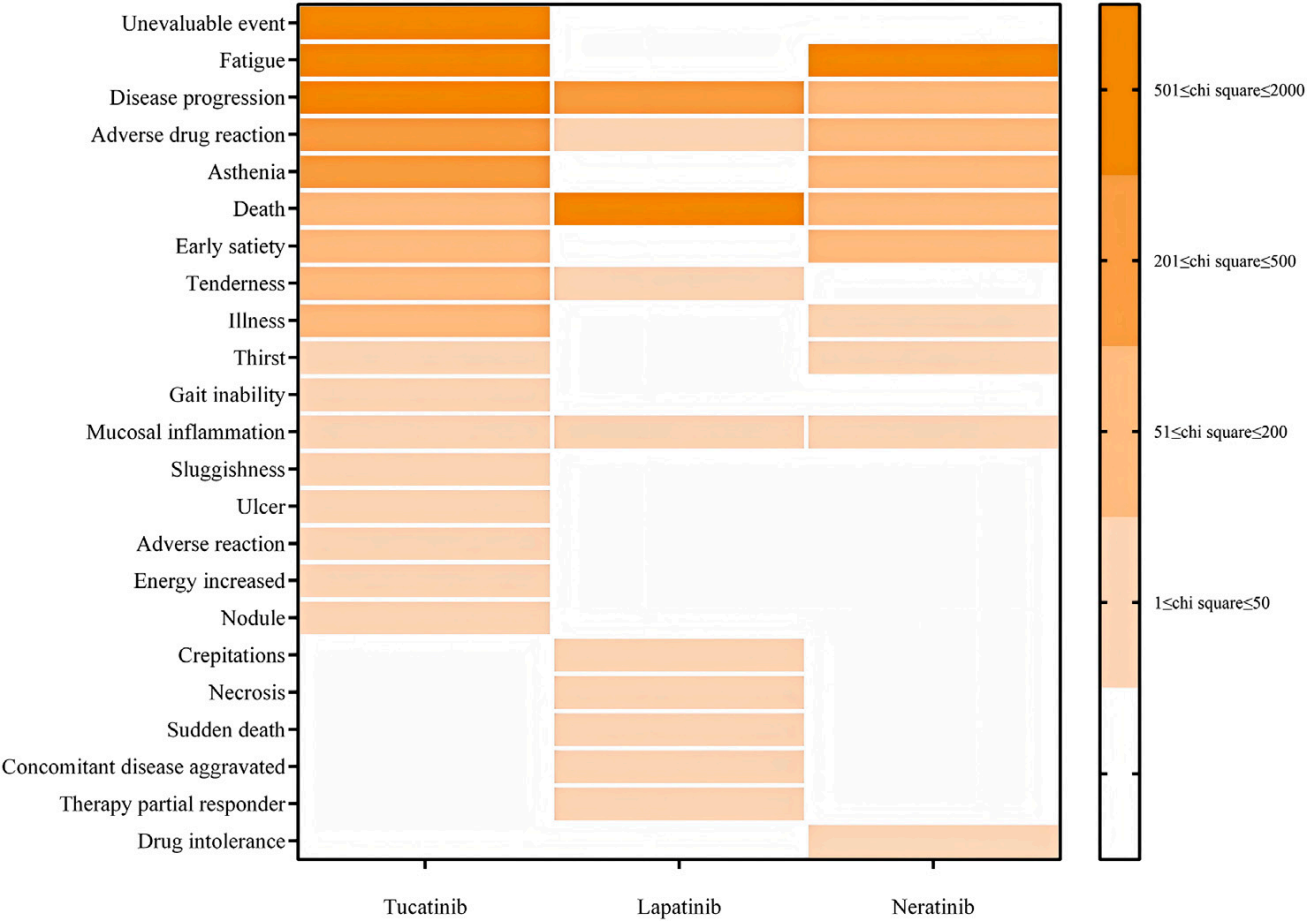
PT distribution for general disorders and administration site reactions.

For general disorders and administration site conditions, Tucatinib was most strongly associated with “Events not assessable” (ROR: 1499.58), followed by fatigue (ROR: 1092.52) and disease progression (ROR: 897.36). Lapatinib was significantly associated with death (ROR: 1415.46), disease progression (ROR: 345.34), and mucosal inflammation (ROR: 14.97). Neratinib exhibited a high prevalence of fatigue (ROR: 1543.41), disease progression (ROR: 173.52), and early satiety (ROR: 158.42) (see Figure 3).

#### 3.3.3 PT distribution in skin and subcutaneous tissue disorders

**FIGURE 4:**
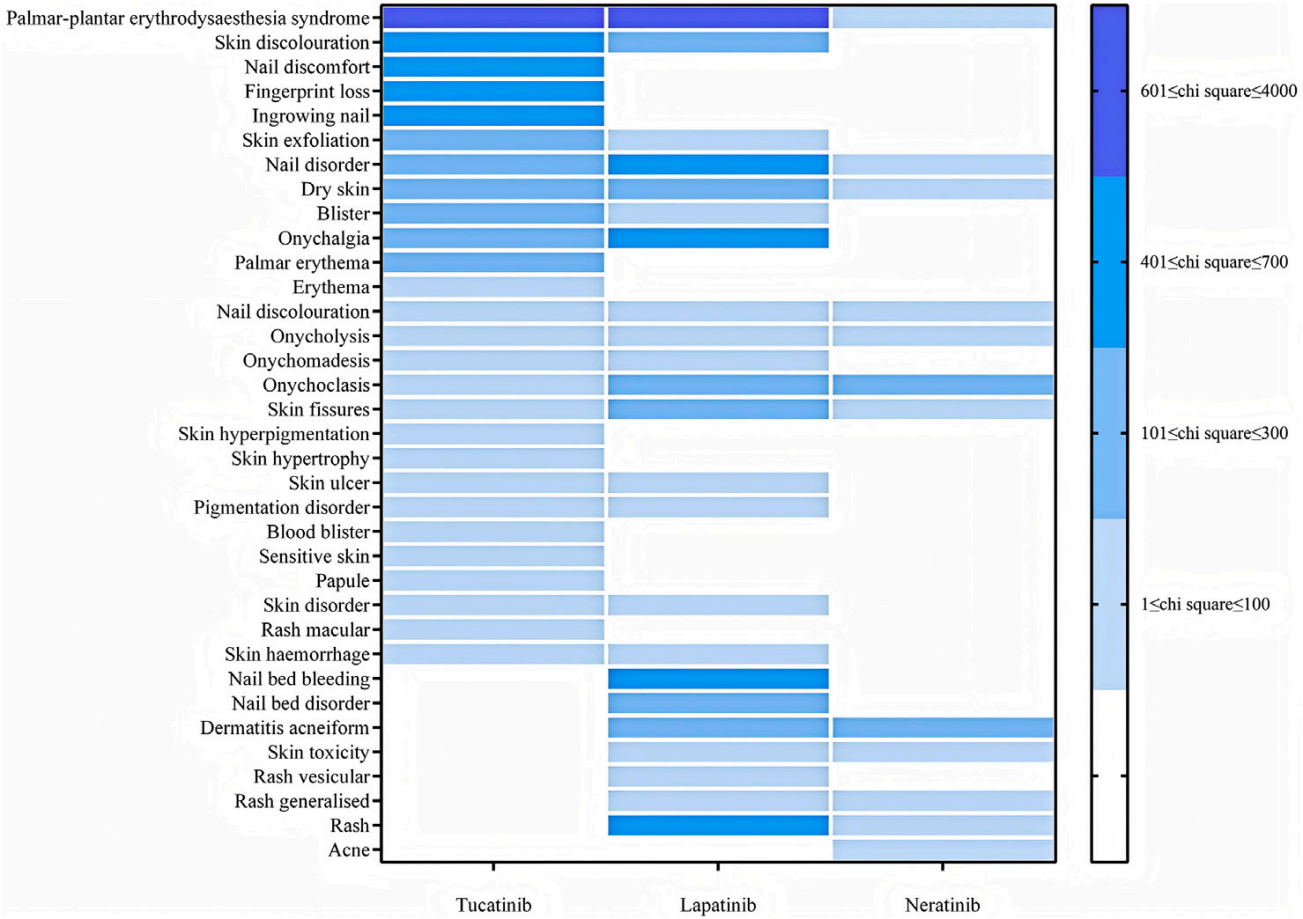
PT distribution for skin and subcutaneous tissue disorders.

For skin and subcutaneous tissue disorders, Tucatinib was most strongly associated with palmar-plantar erythrodysesthesia syndrome (ROR: 3394.03), followed by skin discoloration (ROR: 621.17) and nail discomfort (ROR: 532.41). Lapatinib was significantly associated with palmar-plantar erythrodysesthesia syndrome (ROR: 2146.74), nail pain (ROR: 555.03), and nail bed bleeding (ROR: 484.62). Neratinib exhibited a high prevalence of nail breakage (ROR: 250.41), acneiform dermatitis (ROR: 124.12), and skin fissures (ROR: 84.47) (see Figure 4).

## 4 Discussion

### 4.1 Analysis of basic information on ADE reports

This study presents a detailed analysis of adverse event (ADE) reports for Tucatinib, Lapatinib, and Neratinib based on data from the FAERS database. The analysis examines gender distribution, age distribution, report sources, and geographic trends. Results indicated that most patients were female, consistent with the primary use of these drugs in female-related cancers. The higher proportion of cases with unspecified gender in Neratinib reports, where 96.57% of the gender data was unknown, may result from incomplete reporting. This could be attributed to the reporting preferences of the submitters, who might prioritize patient privacy, leading to the omission of gender information. A small number of male cases were reported, likely associated with rare instances of HER-2 positive breast cancer in men or the use of these drugs in other solid tumors, such as gastric cancer, which is more prevalent in males. Special attention may be required for male patients to monitor potential differences in adverse reactions associated with hormonal variations (Zhou et al., 2021). Regarding age distribution, the majority of patients were aged 18–60 years, though some reports involved children and adolescents. This finding may suggest experimental or personalized use in rare pediatric HER-2-related cancers. Reports involving pediatric patients underscore the need for rigorous safety monitoring, as children exhibit distinct metabolic and drug response mechanisms compared to adults, particularly during long-term TKI therapy. Future studies should collect prospective data and emphasize personalized treatment strategies to more accurately evaluate safety in young patients (Gao et al., 2020). Regarding report sources, most ADEs for Tucatinib and Neratinib were submitted by healthcare professionals, whereas Lapatinib had a higher proportion of reports from non-professionals. This may indicate broader use of Lapatinib among the general population, possibly due to self-medication or self-management in resource-limited areas where patients self-report adverse events (Liu et al., 2021). Geographically, the majority of reports originated from the United States, followed by France, India, and China. This distribution reflects differences in the adoption of these drugs across countries and variations in drug safety monitoring systems. The prevalence of reports from the United States may be attributed to its robust safety monitoring infrastructure and the larger population using these drugs. Reports from France, India, and China highlight a more global adoption of these therapies, with significant contributions from China indicating growing usage and improved drug safety monitoring. Variations across regions underscore the importance of considering factors such as race, healthcare resources, and drug availability, which may influence adverse event incidence (Fan et al., 2024; Chen et al., 2021).

Overall, the gender, age, report sources, and geographic distributions underscore the importance of individualized risk assessments and tailored safety monitoring for diverse patient groups in clinical practice. The broader use of Lapatinib and the instances of self-management highlight the need for personalized decision-making and vigilant monitoring, particularly in areas with limited healthcare resources (Harbeck et al., 2020).

### 4.2 Signal strength and key safety analysis

This study identified high Reporting Odds Ratio (ROR) values for specific adverse event signals, offering valuable insights into clinical risk management. For example, the strong signal for “Gamma ray therapy” associated with Tucatinib may suggest a synergistic effect when combined with other treatments. Meanwhile, the signal for “Nail bed bleeding” associated with Lapatinib highlights a significant risk of skin toxicity. High signals for “Drug titration” and “Breast reconstruction” with Neratinib underscore the need for individualized dose adjustments and highlight the challenges of managing patients undergoing reconstructive surgery (Krop et al., 2021).

Further analysis suggests that the high signal for “Gamma ray therapy” associated with Tucatinib could reflect its specific use alongside radiotherapy. However, the association may simply be due to a synergistic effect with combination therapies, and it currently lacks sufficient validation and in-depth analysis. Therefore, future research should further investigate this synergistic effect and assess its clinical relevance in various treatment combinations. Additionally, the signal for “Congenital malformation of the lung and airway” may point to rare side effects that require attention in specific patient populations (Jones et al., 2022).

The “Nail bed bleeding” signal for Lapatinib underscores severe skin toxicity, potentially associated with its effects on fast-growing cells (Harbeck et al., 2020). Furthermore, the high signals for “Drug titration” and “Breast reconstruction” with Neratinib suggest the need for complex dose adjustments and emphasize challenges faced by patients undergoing long-term treatment after surgery (Chan et al., 2020).

### 4.3 System organ class analysis of ADE signals

As illustrated in Figure 1, the majority of adverse events associated with the three TKIs are categorized under gastrointestinal disorders, general disorders, administration site reactions, and skin and subcutaneous tissue disorders. The subsequent sections provide a detailed analysis of these adverse events and discuss their clinical significance.

#### 4.3.1 Gastrointestinal disorders

Gastrointestinal adverse events represent a significant challenge in the treatment of HER2-positive breast cancer with tyrosine kinase inhibitors (TKIs). Notably, Neratinib exhibits a significantly higher signal for diarrhea compared to other TKIs, indicating a substantial impact on gastrointestinal function that necessitates close monitoring. This effect may result from Neratinib’s nonspecific inhibition of the epidermal growth factor receptor (EGFR), which irritates the gastrointestinal lining, increasing the frequency of diarrhea, nausea, and constipation (Hinnerichs et al., 2022). Effective management strategies for these adverse events include proactive monitoring, preventive use of antidiarrheal medications, dietary guidance, and dose adjustments to alleviate symptoms and improve patient comfort (Saura et al., 2020; Crown et al., 2021). Tucatinib and Lapatinib are also associated with gastrointestinal side effects, though these tend to be less severe than those observed with Neratinib. For these drugs, early intervention and personalized management are crucial to reducing the risk of adverse effects and maintaining treatment adherence (Murthy et al., 2020; Cameron et al., 2010).

#### 4.3.2 General disorders and administration site reactions

General disorders and administration site reactions, including fatigue and indicators of disease progression, were prevalent across all three drugs. These findings underscore the importance of vigilant health monitoring, particularly for patients receiving long-term treatment. Fatigue, a common consequence of cancer therapy, is influenced by multiple factors, including drug toxicity, the cancer itself, and the patient’s physical and psychological state (Abrahams et al., 2016). In clinical practice, fatigue management includes supportive care, psychological counseling, and personalized treatment adjustments (Bower, 2014). Reports of disease progression suggest that these drugs may be insufficiently effective for certain patients, highlighting the need for regular evaluations and treatment adjustments (Diéras et al., 2017). The higher mortality rate reported, particularly in the Lapatinib group, indicates a substantial burden of adverse effects among high-risk patients, necessitating thorough patient selection and vigilant monitoring to mitigate risks and optimize outcomes (Cameron et al., 2010).

#### 4.3.3 Skin and subcutaneous tissue disorders

Adverse events affecting the skin and subcutaneous tissues represent significant concerns for all three TKIs. Palmar-plantar erythrodysesthesia syndrome (PPES) is particularly prevalent among patients receiving Tucatinib or Lapatinib (Montemurro et al., 2019). This reaction is believed to result from the inhibition of epidermal keratinocytes by these drugs (Sadeghi et al., 2020) To alleviate symptoms, patients are advised to use topical moisturizers, minimize excessive friction, and adjust doses as necessary (Duvic et al., 2018). Early identification and supportive care are essential for effectively managing hand-foot syndrome and preventing treatment interruptions (Razonable and Aithal, 2018). Other adverse events, such as skin discoloration and nail-related issues, are also frequent and may adversely impact patients’ quality of life or cause emotional distress (Dirks et al., 2021). Healthcare providers should reassure patients that these side effects are typically reversible and manageable, which can help sustain adherence to treatment (Fischer et al., 2020). Furthermore, the high incidence of acneiform dermatitis observed in Neratinib-treated patients suggests an inflammatory response. Managing this condition may involve topical anti-inflammatory agents and meticulous skin care (Sun et al., 2021).

### 4.4 Future research directions

Building upon the findings from this study, future research should focus on further enhancing our understanding of the safety profiles of Tucatinib, Lapatinib, and Neratinib. The potential synergistic effect of Tucatinib when used in combination with radiotherapy is particularly worth exploring. Although a strong signal was observed for “Gamma ray therapy,” this association needs to be validated in clinical settings to assess whether this potential interaction significantly impacts treatment outcomes. Additionally, further investigations should address the rare side effects observed with Tucatinib, such as “Congenital malformation of the lung and airway.” These effects should be studied in targeted patient populations to better understand the underlying mechanisms and predisposing factors, which could lead to more tailored risk management strategies. The complexity of managing dose adjustments and long-term therapy in patients undergoing reconstructive surgery highlights the importance of personalized treatment protocols, particularly for Neratinib. Future studies could include detailed assessments of the pharmacokinetics and pharmacodynamics of Neratinib in different patient groups, providing insights to optimize treatment regimens. Finally, expanding this research to include a more diverse global population, as well as additional HER-2 positive cancer types, would further validate these findings and contribute to a broader clinical application. This would not only improve patient safety but also enhance treatment outcomes.

## 5 Research limitations

This study has several limitations. The FAERS database, while a valuable resource for pharmacovigilance, is subject to underreporting and voluntary reporting biases. Such biases can lead to an incomplete representation of adverse events and affect the reliability of the findings. Additionally, since the data are collected from various sources, including healthcare providers and patients, there is a risk of data inconsistency or inaccuracies. Moreover, the exclusion of duplicate reports was based on a deduplication process, which may not entirely eliminate errors. Finally, the results of this study should be interpreted with caution due to the inherent limitations of retrospective data analysis and the absence of a direct causal relationship between the drugs and adverse events.

## 6 Conclusion

This study presents a systematic analysis of adverse events associated with Tucatinib, Lapatinib, and Neratinib in the treatment of HER-2 positive breast cancer, utilizing FAERS database data to elucidate the key safety characteristics and toxicity profiles of these drugs. Gastrointestinal disorders, general conditions, and skin and subcutaneous tissue disorders were identified as the primary areas of concern across the three TKIs. The gastrointestinal side effects of Neratinib warrant special attention, while the skin and systemic reactions associated with Lapatinib represent distinct risks. Future research should focus on further validating these findings, identifying specific risk factors in diverse patient subgroups, and evaluating the effectiveness of various management strategies. This approach aims to optimize therapeutic benefits for patients while minimizing adverse effects, ultimately enhancing overall quality of life and treatment adherence.

## Data Availability

Publicly available datasets were analyzed in this study. This data can be found here: FDA Adverse Event Reporting System (FAERS) (https://fis.fda.gov/extensions/FPD-QDE-FAERS/FPD-QDE-FAERS.html).
